# DLA Class II Alleles and Haplotypes Are Associated with Risk for and Protection from Chronic Hepatitis in the English Springer Spaniel

**DOI:** 10.1371/journal.pone.0042584

**Published:** 2012-08-01

**Authors:** Nicholas H. Bexfield, Penny J. Watson, Jesús Aguirre-Hernandez, David R. Sargan, Laurence Tiley, Jonathan L. Heeney, Lorna J. Kennedy

**Affiliations:** 1 Department of Veterinary Medicine, University of Cambridge, Cambridge, United Kingdom; 2 Centre for Integrated Genomic Medical Research, University of Manchester, Manchester, United Kingdom; University of Montreal, Canada

## Abstract

Chronic hepatitis (CH) is common in dogs in the United Kingdom. An increased prevalence of the disease is seen in the English Springer spaniel (ESS), and this breed suffer from a severe form with young to middle aged female dogs being predisposed. The disease shares histological features with those of human viral hepatitis, although the specific aetiological agent has not yet been identified. The aim of the current study was to investigate whether dog leucocyte antigen (DLA) class II alleles and haplotypes are associated with susceptibility/resistance to CH in the ESS. Sequence-based genotyping of the polymorphic exon 2 from DLA-DRB1, -DQA1 and -DQB1 class II loci were performed in 66 ESSs with CH and 84 healthy controls. There was a significant difference in the distribution of the protective alleles DRB1*00501 (3.0% vs. 12.0%, odds ratio [OR] = 0.23, 95% confidence interval [CI] = 0.06–0.74) and DQB1*00501 (3.8% vs. 12.0%, OR = 0.29, 95% CI = 0.09–0.85) between cases and controls. The haplotype DLA-DRB1*00501/DQA1*00301/DQB1*00501 was present in 11.9% of controls and 3.0% of cases and was significantly associated with protection against disease development (OR = 0.26, 95% CI = 0.08–0.80). There was a significant difference in the distribution of the risk alleles DRB1*00601 (14.4% vs. 6.5%, OR = 2.40, 95% CI = 1.10–5.63) and DQB1*00701 (14.4% vs. 6.5%, OR = 2.40, 95% CI = 1.10–5.63) between cases and controls. A risk haplotype (DLA-DRB1*00601/DQA1*005011/DQB1*00701) was present in 14.4% of cases and 6.5% of controls and conferred an elevated risk of developing CH with an OR of 3.13 (95% CI = 1.20–8.26). These results demonstrate that DLA class II is significantly associated with risk and protection from developing CH in ESSs.

## Introduction

Canine chronic hepatitis (CH) is common in the United Kingdom with a prevalence of 12% at post mortem [1]. However, the aetiology of most cases of canine CH remains unknown [2]. Known causes identified in a small number of cases include canine adenovirus type I (CAV-1) [3], bacteria including leptospires [4] and *Helicobacter* spp. [5], and several toxins and drugs [6]. Defects in copper metabolism have been described in several breeds [7], and some dogs accumulate alpha-1 antitrypsin in hepatocytes leading to cell death [8]. To date, studies have failed to conclusively demonstrate a primary immune-mediated aetiology [9], [10].

An increased prevalence of CH occurs in the English Springer spaniel (ESS) [11], suggesting a genetic predisposition to disease. The ESS suffers from a more aggressive form of CH than other breeds with a median survival of just over six months [11], compared to 19 months in a variety of other breeds of dog with CH [12]. The pathogenesis of the disease is incompletely elucidated, but is of suspected viral aetiology as it shares histological features with those of chronic viral hepatitis in humans [11], [13], [14].

The canine major histocompatibility complex (MHC), referred to as the dog leucocyte antigen (DLA) system, plays a central role in the control of the immune system and comprises of three regions known as class I, II and III. The first two regions are involved in the regulation and presentation of self and non-self antigens to the immune system. The class I region contains one highly polymorphic gene called DLA-88 plus several other genes [15], [16]. The class II region includes four functional genes; DLA-DRA1 which appears monomorphic and DLA-DRB1, DQA1 and DQB1 which are highly polymorphic [15], [16]. The full extent of their polymorphism has not yet been determined. There are currently 106 DLA-DRB1, 26 DLA-DQA1 and 62 DLA-DQB1 alleles identified in the dog and other closely related canids [17]. Furthermore, particular DLA class II allelic forms of each locus tend to be found together on the same chromosome, or haplotypes, more frequently than expected from their own individual gene frequencies. This linkage disequilibrium, or non-random association of alleles at adjacent MHC loci, results in conserved haplotype combinations which often have a restricted breed distribution [18]. Over 144 different three-locus, DLA-DRB1/DQA1/DQB1, haplotypes have been identified in more than 80 breeds of dog [18]. Although there is often a lack of within breed variability in MHC alleles expressed in pedigree dogs, the ESS shows a slightly above average diversity of MHC alleles [18], [19]. Because dogs and humans share a similar set of orthologous genes, are affected by diseases of similar aetiology and live in the same environment, the dog is a useful model for studies on genetic diseases [20], [21], [22].

MHC genes, known as the human leucocyte antigens (HLA), are important in determining susceptibility to autoimmune, metabolic and infectious diseases in man [23], [24], [25]. For instance several studies have reported the influence of MHC genotype on the outcome of viral infections, such as reported in HIV-1 infected persons [26], [27], [28]. In humans there are also important associations between HLA polymorphisms and clinical outcome after infection with the hepatotrophic viruses, hepatitis B (HBV) and C (HCV). In HCV infection, the most profound association has been with natural killer cell immunoglobulin-like receptors (KIRs) that recognise HLA-C [29], [30], while in HBV infected individuals HLA class II alleles have been associated with both protection from, and risk of disease progression [31], [32].

To date, the role of DLA in disease susceptibility has largely been established for canine immune-mediated diseases, although an association between DLA haplotypes and other canine non immune-mediated diseases including generalised demodicosis [33], anal gland carcinoma [34], and cranial cruciate ligament rupture [35] has also been identified. Only one study has also examined the possible association between DLA class II genes and canine hepatitis in the Dobermann Pincher, a disease of suspected autoimmune aetiology [36].

The aim of this study was to determine whether DLA class II alleles and haplotypes were associated with CH in the ESS.

## Results

The study cohort of dogs with CH comprised 47 females and 19 males, and the median age was three years 11 months (range, seven months to eight years one month). The median age of the control dogs was nine years eight months (range eight years to 14 years one month) and there were 55 females and 29 males.

DLA class II alleles and haplotypes were assigned to all dogs with CH and control dogs. Within this group of ESS we identified ten DLA-DRB1 alleles, six DLA-DQA1 alleles and eight DLA-DQB1 alleles (Table 1). Of the DRB1 alleles, DRB1*01501 was the most common allele with a frequency of 39.3%, and the other nine alleles had frequencies between 0.4–24.3%. 40.0% of ESSs had allele DQA1*00601 and the other alleles ranged in frequency between 1.3–33.0%. Of the DQB1 alleles, DQB1*01303 was the most common (38.3%) and the others had frequencies between 1.3–22.7%. For DLA-DRB1, there was a significant difference in the distribution of the alleles DRB1*00501 (3.0% vs. 12.0%, odds ratio [OR] = 0.23, 95% confidence interval [CI] = 0.06–0.74) and DRB1*00601 (14.4% vs. 6.5%, OR = 2.40, 95% CI = 1.10–5.63) between cases and controls. For DLA-DQB1, two alleles, namely DQB1*00501 (3.8% vs. 12.0%, OR = 0.29, 95% CI = 0.09–0.85) and DQB1*00701 (14.4% vs. 6.5%, OR = 2.40, 95% CI = 1.10–5.63) had a significantly different distribution between cases and controls.

**Table 1:** Frequencies of DLA class II alleles in 66 ESSs with CH and 84 healthy controls.

DLA-DRB1	Cases (frequency %) n = 132	Controls (frequency %) n = 168	Total (frequency %) n = 300	OR	95% CI	Raw P value*
00101	11 (8.3)	11 (6.5)	22 (7.3)			
00501	**4 (3.0)**	**20 (12.0)**	**24 (8.0)**	**0.23**	**0.06–0.74**	
00601	**19 (14.4)**	**11 (6.5)**	**30 (10.0)**	**2.40**	**1.10–5.63**	
00901	1 (0.8)	3 (1.8)	4 (1.3)			
01201	30 (22.7)	43 (25.6)	73 (24.3)			
01501	50 (37.9)	68 (40.5)	118 (39.3)			
01502	2 (1.5)	0	2 (0.7)			
02001	14 (10.6)	11 (6.5)	25 (8.3)			
02301	1 (0.8)	0	1 (0.4)			
012v	0	1 (0.6)	1 (0.4)			
Clump test 1 for locus (chi squared emulation, all alleles)						0.012
Clump test 2 for locus (chi squared emulation, after grouping alleles with small values together)						0.014

DLA-DQA1	Cases (frequency %) n = 132	Controls (frequency %) n = 168	Total (frequency %) n = 300	OR	95% CI	Raw P value*
00101	1 (0.8)	3 (1.8)	4 (1.3)			
00201	11 (8.3)	11 (6.5)	22 (7.3)			
00301	5 (3.8)	20 (12.0)	25 (8.3)	0.29	0.09–0.85	
00401	44 (33.3)	55 (32.7)	99 (33.0)			
00601	52 (39.4)	68 (40.5)	120 (40.0)			
005011	19 (14.4)	11 (6.5)	30 (10.0)	2.40	1.10–5.63	
Clump test 1 for locus (chi squared emulation, all alleles)						0.041 (NS)
Clump test 2 for locus (chi squared emulation, after grouping alleles with small values together)						0.033 (NS)

DLA-DQB1	Cases (frequency %) n = 132	Controls (frequency %) n = 168	Total (frequency %) n = 300	OR	95% CI	Raw P value*
00301	26 (19.7)	42 (25.0)	68 (22.7)			
00501	**5 (3.8)**	**20 (12.0)**	**25 (8.3)**	**0.29**	**0.09–0.85**	
00701	**19 (14.4)**	**11 (6.5)**	**30 (10.0)**	**2.40**	**1.10–5.63**	
008011	1 (0.8)	3 (1.8)	4 (1.3)			
01303	49 (37.1)	66 (39.3)	115 (38.3)			
013017	6 (4.5)	0	6 (2.0)			
02002	23 (17.4)	24 (14.2)	47 (15.7)			
02301	3 (2.3)	2 (1.2)	5 (1.7)			
Clump test 1 for locus (chi squared emulation, all alleles)						0.003
Clump test 2 for locus (chi squared emulation, after grouping alleles with small values together)						0.010

Altogether, 10 DRB1 alleles, six DQA1 alleles and eight DQB1 alleles were found in the population. The alleles DRB1*00601and DQB1*00701 were observed in a higher frequency in cases while the alleles DRB1*00501and DQB1*00501 were more frequent in controls. Numbers in bold indicate a significant difference between cases and controls. NS; not significant.

* After Bonferroni adjustment, significance level would be p<0.017.

There were a total of 11 different DLA-DRB1/DQA1/DQB1 haplotypes with frequencies >1% (Table 2). In the controls there were two haplotypes with frequencies around 25%, two with frequencies between 10–15%, three with frequencies of 6.5%, two with frequencies between 1–2% and one with a frequency of <1%. In the affected cases there were three haplotypes with frequencies between 15–20%, three with frequencies between 8–15%, three with frequencies between 1–5%, and three with frequencies <1%. In ESSs, two haplotypes differed markedly in frequency between cases and controls (Table 2). The haplotype DLA-DRB1*00601/DQA1*005011/DQB1*00701 (haplotype four) occurred in 14.4% of the cases compared with 6.5% of the controls (OR = 3.13, 95% CI = 1.20–8.26) and was defined as a risk-haplotype. One affected and two control dogs were homozygous for this risk haplotype. A lower frequency of CH was found in ESSs carrying haplotype DLA-DRB1*00501/DQA1*00301/DQB1*00501 (haplotype eight). This haplotype was found in 11.9% of the controls and 3.0% of the cases (OR = 0.26, 95% CI = 0.08–0.80) and thus is significantly associated with protection against disease development. No affected or control dog was homozygous for this protective haplotype.

**Table 2:** Frequencies of three locus DLA class II haplotypes in 66 ESSs with CH and 84 healthy controls.

				Cases		Controls				
Number	DRB1	DQA1	DQB1	Number n = 132	Frequency (%)	Number n = 168	Frequency (%)	Odds ratio	95% CI	P-value
1	01501	00601	00301	26	19.7	42	25.0			
2	01201	00401	01303	24	18.2	43	25.6			
3	01501	00601	02002	23	17.4	24	14.3			
4	00601	005011	00701	**19**	**14.4**	**11**	**6.5**	**3.13**	**1.20–8.26**	
5	02001	00401	01303	14	10.6	11	6.5			
6	00101	00201	01303	11	8.3	11	6.5			
7	01201	00401	013017	6	4.5	0	0.0			
8	00501	00301	00501	**4**	**3.0**	**20**	**11.9**	**0.26**	**0.08–0.80**	
9	01502	00601	02301	2	1.5	0	0.0			
10	00901	00101	008011	1	0.8	3	1.8			
11	01501	00601	02301	1	0.8	2	1.2			
	Other single haplotypes			1	0.8	1	0.6			
Clump test 1: cases v controls (chi squared emulation, all haplotypes										0.0017
Clump test 2 cases v controls (chi squared emulation, after grouping haplotypes with small values together)										0.0055

A total of 11 different haplotypes with frequencies >1% were identified. DLA-DRB1*00601/DQA1*005011/DQB1*00701 (haplotype four) had an increased frequency in cases and DLA-DRB1*00501/DQA1*00301/DQB1*00501 (haplotype eight) was significantly more frequent in controls, both numbers shown in bold. A p value for significance was set at 0.05 for comparison of haplotype frequencies.

## Discussion

The results of this study demonstrate a significant association between DLA class II and CH in the ESS, and moreover that this may represent an important immunological risk factor for the development, or the progression and persistence, of the disease. This apparent genetic component to CH has been previously suspected in view of the predilection of this disease to occur in particular breeds including the ESS and reports of familial occurrence [37].

The present, and the majority of previous studies, have used a candidate gene approach to examine DLA class II genes. While the typing of class II genes using a sequence-based method is relatively simple, typing of the class I genes is more difficult as it requires amplification and cloning [38]. Moreover, comparatively less is known about DLA class I genes. As a first step in understanding the genetics of CH in ESS, we therefore chose to analyse class II genes, although future studies could also study the association with class I genes. Due to the high linkage disequilibrium in the MHC class II region, and the extensive polymorphism in exon two (which encodes the antigen binding domain), genetic typing of this exon was used in an attempt to detect most of the variation in the locus. The direct sequencing of the class II region yielded the characterized DLA-DRB1/DQA1/DQB1 alleles and haplotypes [17] and was used in the investigation of disease associations [39]. Because very few dogs were homozygous, we calculated haplotype and allele frequencies, rather than the number of dogs with each haplotype or allele. When performing genetic typing, all control dogs, and all dogs with CH for whom pedigree information was available (45/66) were unrelated at the grandparent level. However, it is possible that if some of the remaining dogs in the CH group were closely related, this could lead to an overrepresentation of alleles and haplotypes in this group.

There was a significant difference in the distribution of the alleles DRB1*00501, and DQB1*00501 between cases and controls, with these alleles offering protection from disease. A third allele, DQA1*00301, also approached significance as a protective allele. Haplotype eight appeared to confer protection against CH and this was identified with a frequency of 3.0% in cases and 11.9% in controls (OR = 0.26, 95% CI = 0.08–0.80). This haplotype is relatively common in the ESS, being found in 8.3% of ESS [18]. However, this haplotype was not found in Dobermann Pinschers with hepatitis [36] nor in ESSs with IMHA [40].

There was a significant difference in the distribution of the alleles DRB1*00601 and DQB1*00701 between cases and controls and these were risk alleles. A third allele, DQA1*005011, also approached significance as a risk allele. A significant association between haplotype four and the presence of CH was also observed (OR = 3.13, 95% CI = 1.20–8.26), suggesting that this haplotype is a potential risk haplotype for disease development. Only one affected dog was homozygous for the risk haplotype. Haplotype four is one of the most widespread of the DLA haplotypes across all dogs, having being found in 40 of 88 breeds [18] with a reported frequency of 8.7% [19]. While this haplotype is very common in Cocker Spaniels (>60% of chromosomes in several studies), it also reaches moderate frequency (17%) in ESSs [18]. In the present study haplotype four had a frequency of 10% in all ESSs. Haplotype four has been shown to be associated with other canine diseases, including anal gland carcinoma in the English Cocker Spaniel [34]. In primary IMHA this haplotype was also present in 30.3% of all cases compared to 19.1% of controls and was one of two potential risk haplotypes [40]. However, when individual breeds with primary IMHA were examined, this haplotype was not increased in affected ESSs.

Two control dogs were homozygous for the risk haplotype, but had no evidence of disease. This is likely to be due to the fact that CH is a complex trait with an additional environmental insults leading to disease development. The most likely reason for the lack of homozygosity for the protective haplotype among the control dogs is the relatively limited number of animals studied. Even with a heterozygote frequency of 11.9% in the control population, one would only expect one homozygote by chance alone, with no homozygotes being the second most likely outcome. However, it is also possible that there are also detrimental health implications for homozygotes, therefore removing them from the population.

MHC class II antigens mainly determine which antigenic peptides an individual is able to present to CD4^+^ T-lymphocytes in order to stimulate an immune response [41]. Expression of MHC class II is normally restricted to professional antigen-presenting cells, but it can be induced in other cell types by autoimmune, infectious or neoplastic diseases [42]. For example, epithelium-like cells can be induced to express MHC class II molecules upon exposure to inflammatory cytokines [43], [44], or viral antigens [45], [46]. Human hepatocytes frequently exhibit aberrant MHC class II expression in viral hepatitis [47], [48], and it has also been shown that canine hepatocytes can express MHC class II during inflammation [49]. The induction of MHC class II molecules on such cells during viral infection likely plays an important role in the protective immune response of host against the virus by lysis of infected cells. Alternatively, this may lead to the development of infection-associated immunopathology by lysis of both infected and neighbouring cells that passively acquire released viral antigen.

Polymorphisms of the human immune regulatory genes, or HLA class I and II molecules, are important in influencing the host's ability to present or react to viral antigens [50]. In the case of human viral hepatitis, certain HLA haplotypes are strongly associated with the progression of liver injury following infection with HCV, whereas other are associated with HCV clearance or a lower risk of developing liver injury [29], [30], [51], [52], [53]. There are associations between certain HLA class II alleles and clearance of HBV and also an increase in viral persistence and the development of chronic liver disease [31], [32], [54]. CH in the ESS is of suspected viral aetiology as it shares histological features to those of chronic viral hepatitis in humans including predominantly lymphocytic inflammation and necrosis and apoptosis in areas of inflammation [11], [13], [14]. The canine MHC also plays a central role in the control of the immune response to infectious agents. Selective inbreeding has, however, led to a restriction of DLA haplotypes in most breeds, which in turn will influence the susceptibility to infectious diseases. We hypothesise that the highly polymorphic DLA genes are involved in increased or decreased susceptibility to CH in the ESS, although genetic and other environmental factors are also likely involved in disease development. Akin to humans with natural HCV infection where some individuals are not infected despite high levels of exposure [55], others clear virus following infection, and other have progressive disease resulting in CH [56], a similar outcome may occur in the ESS exposed to a putative virus. Studies utilizing modern molecular techniques to identify the novel viral agent causing CH in the ESS are ongoing [57].

The only other study to investigate the possible association between DLA class II genes and canine hepatitis was performed in the Dobermann Pinscher [36]. This study identified DLA-DRB1*00601 as a risk allele for the disease, with all affected, and 56.8% of control animals homozygous for this allele. This allele was found in combination with DLA-DQA1*00401/DQB1*01303, and 94.6% of affected Dobermann Pinschers were homozygous for this risk haplotype. In the present study, this haplotype was not found in any affected or control ESS. However, the allele DLA-DRB1*00601 was present in 14.4% cases and 6.5% controls, always in combination with DQA1*005011/DQB1*00701 (haplotype four). Although the aetiology of hepatitis in the Dobermann Pincher is not known, an autoimmune aetiology is postulated. A T-cell mediated response is activated in genetically predisposed individuals and affected hepatocytes express MHC class II antigens [49]. Aberrant MHC class II expression is seen in human autoimmune liver disease, although this also occurs in virally infected hepatocytes [58]. In Dobermann Pincher hepatitis, MHC class II expression has been shown to be persistent and increased on the hepatocyte membrane during disease progression [49]. Although no studies have been performed to determine if CH in the ESS has an autoimmune aetiology, features such as an abundance of plasma cells and multi nucleated giant cells, common in human autoimmune parenchymal liver disease [13], are not apparent [11]. In addition, ESS with CH exhibit a poor response to corticosteroids [59] and do not have elevated serum globulins [11], a hallmark of human autoimmune hepatitis [60].

In conclusion, we have identified two alleles and one haplotype that appear to protect against the development of CH in the ESS, and two alleles and one haplotype that appears to confer risk of disease development. However, it is likely that the disease has a complex pathogenesis, whereby multiple genetic and environmental components interact to trigger and drive continued disease progression. The fact that relatively few major human genes have been identified in several genome-wide linkage scans for bacterial, parasitic and viral infectious diseases, supports the view that the genetic susceptibility is widely distributed among numerous polygenes [61]. Further identification of additional genetic risk factors for CH in the ESS is currently being performed by genome-wide association analysis using canine high density SNP arrays [62]. The results of the present study are, however, novel and likely to be of comparative value in understanding the aetiology of CH in other breeds of dog. Moreover, our findings could be used to assist breeding practices by increasing the frequency of the protective haplotype in an effort to reduce the incidence of CH in ESSs.

## Materials and Methods

### Study population

Ethylenediaminetetraacetic acid (EDTA) anticoagulant blood samples from 66 ESSs with CH were collected between 2006 and 2011. Samples had been submitted for haematological analysis to the Central Diagnostic Services Laboratory, University of Cambridge from the Queen's Veterinary School Hospital and other external practices in the UK. The diagnosis of CH was based on consistent clinical signs, the presence of elevated liver enzymes and confirmed by histological examination of liver tissue using standardised criteria for diagnosis [63]. It was not possible to definitively investigate the relatedness in this population as pedigree information was not available for all dogs. Pedigree information was available for 45 dogs and all were unrelated at least to the grandparental level. Analysis of the date of birth of the remaining dogs confirmed that these animals were not siblings.

Control EDTA blood samples were obtained from residual blood samples collected from 84 ESS aged eight years or over between 2009 and 2011. Since CH occurs in young to middle aged ESSs [11], we chose older dogs to use as the control group. No dog had clinical signs of liver disease and all dogs had normal liver enzymes measured at the time of blood collection. Pedigree information was available for all control dogs and all were unrelated at least to the grandparental level. All blood was stored at −20°C for subsequent analysis of DLA genes.

### Ethics statement

All samples consisted of residual blood remaining after diagnostic testing and were collected in accordance with guidelines of the Royal College of Veterinary Surgeons, UK and the Veterinary Surgeons Act 1966. For this reason ethical committee approval was not required. All samples were collected with informed and written owner consent.

### MHC genotyping for DLA-DRB1, DQA1 and DQB1

DNA was extracted from blood samples using the QIAamp DNA Blood Midi Kit (Qiagen, Crawley, UK) according to the manufacturer's instructions. DNA concentration was measured using a fluorescence-based method (Quant-iT PicoGreen dsDNA Assay, Life Technologies, Paisley, UK), and samples were normalised to 20 ng/µl. Dogs were characterised for three DLA class II loci using sequence based typing [64], [65]. Polymerase chain reactions (PCR) amplification was performed with 25 ng genomic DNA in a 25 µl reaction containing 1× PCR buffer (Qiagen), Q solution (Qiagen), final concentration of 0.1 µM each primer, 200 µM each dNTP and 2.5units Taq polymerase (HotStar Taq, Qiagen). A negative control containing no DNA template was included in each run of amplification to identify possible contamination. The primers used for DLA-DRB1 were forward DRBIn1: CCG TCC CCA CAG CAC ATT TC and reverse DRBIn2-T7: TAA TAC GAC TCA CTA TAG GG TGT GTC ACA CAC CTC AGC ACC A. The primers used to amplify DLA-QA1 were forward DQAIn1: TAA GGT TCT TTT CTC CCT CT and reverse DQAIn2: GGA CAG ATT CAG TGA AGA GA. The primers used to amplify DLA-DQB1 were forward DQB1B-T7: TAA TAC GAC TCA CTA TAG GG CTC ACT GGC CCG GCT GTC TC and reverse DQBR2: CAC CTC GCC GCT GCA ACG TG. The T7 tails are underlined. All primers were intronic and locus specific, and the product sizes were DLA-DRB1 (303 bp), DQA1 (345 bp) and DQB1 (300 bp). A standard touchdown PCR protocol was employed for all amplifications which consisted of an initial 15 min at 95°C, 14 touchdown cycles of 95°C for 30 s, followed by 1 min annealing, starting at 62°C (DRB1), 54°C (DQA1), 73°C (DQB1) and reducing by 0.5°C each cycle, and 72°C for 1 min. Then, 20 cycles of 95°C for 30 s, 55°C (DRB1), 47°C (DQA1) and 66°C (DQB1) for 1 min, 72°C for 1 min and a final extension at 72°C for 10 min were performed.

To check for the presence of a product, 5 µl amplified PCR products were resolved by electrophoresis in a 2% agarose gel, stained with ethidium bromide and viewed under ultraviolet transillumination. Prior to sequencing, all samples were purified as follows: 2units of shrimp alkaline phosphatase (Amersham, Little Chalfont, UK), and 10units of Exo1 (New England Biolabs, Hitchin, UK) were added to 5 µl of PCR product. The mixture was incubated for 1 hour at 37°C, then for 15 min at 80°C. Cycle sequencing (using T7 for DLA-DRB1 and DQB1, and DQAIn2 for DLA-DQA1) was performed using Big Dye Terminator V3 (Life Technologies), and samples were sequenced on an Applied Biosystems 373 Genetic Analyzer. Sequencing data was analysed using SBTengine (GenDX, Netherlands).

### Haplotype assignment and statistical methods

Three-locus, DLA-DRB1/DQA1/DQB1, haplotypes were identified by following a sequential analytical process. First, all dogs that were homozygous at all three loci were selected, and from these several different DLA-DRB1/DQA1/DQB1 haplotype combinations were identified. Dogs that were homozygous at only two loci were then selected. From these dogs, many of the previous haplotypes were confirmed and also several additional haplotypes were identified. The remaining dogs were examined using the haplotype data already identified, and haplotypes were assigned to each of these dogs. From these dogs further possible haplotypes were identified. Allele and haplotype frequencies in cases and controls were compared using the program Clump v22 [66], to perform chi squared emulation for 2×n contingency tables (where n is the number of alleles at each locus or the number of haplotypes being scored), using 100,000 trials at each locus or for the haplotype data. The p value used for significance was set at 0.017 for comparison of allele frequencies as there were a total of three tests per locus. A p value for significance was set at 0.05 for comparison of haplotype frequencies. Where differences were detected, OR and 95% CIs were calculated for disease association of individual alleles or haplotypes.
